# Association between bilirubin and cardiovascular disease risk factors: using Mendelian randomization to assess causal inference

**DOI:** 10.1186/1471-2261-12-16

**Published:** 2012-03-14

**Authors:** Patrick F McArdle, Brian W Whitcomb, Keith Tanner, Braxton D Mitchell, Alan R Shuldiner, Afshin Parsa

**Affiliations:** 1 Division of Endocrinology, Diabetes and Nutrition, University of Maryland School of Medicine, 660 West Redwood Street, Rm. 492, Baltimore, MD 21201, USA; 2 Division of Biostatistics and Epidemiology, School of Public Health and Health Science, University of Massachusetts, Amherst, MA, USA; 3 Geriatric Research and Education Clinical Center, Veterans Administration Medical Center, Baltimore, MD, USA; 4 Division of Nephrology, University of Maryland School of Medicine, Baltimore, MD, USA

**Keywords:** Bilirubin, Mendelian randomization, Cardiovascular disease

## Abstract

**Background:**

Elevated serum bilirubin has been associated with reduced risk of cardiovascular disease (CVD). However, serum bilirubin is also related with several potential confounders related to CVD, such as obesity. Mendelian randomization has been proposed as a method to address challenges to validity from confounding and reverse causality. It utilizes genotype to estimate causal relationships between a gene product and physiological outcomes. In this report, we demonstrate its use in assessing direct causal relations between serum bilirubin levels and CVD risk factors, including obesity, cholesterol, measures of vascular function and blood pressure.

**Methods:**

Study subjects included 868 asymptomatic individuals. Study subjects were genotyped at the UGT1A1*28 locus, which is strongly associated with bilirubin levels.

**Results:**

Serum bilirubin levels were inversely associated with levels of several cardiovascular disease risk factors, including body mass index (*p *= 0.003), LDL (*p *= 0.0005) and total cholesterol (*p *= 0.0002). In contrast, UGT1A1*28 genotype, a known cause of elevated bilirubin levels, was not significantly associated with any of these traditional CVD risk factors. We did observe an association between genotype and brachial artery diameter (*p *= 0.003) and cold pressor reactivity (*p *= 0.01).

**Conclusions:**

Our findings imply that the observed association of serum bilirubin levels with body mass index and cholesterol are likely due to confounding and suggest that previously established CVD benefits of increased bilirubin may in part be mediated by the early regulation of vascular structure and reactivity.

## Background

Bilirubin is a metabolic byproduct of the breakdown of hemoglobin degradation which itself must be metabolized for appropriate excretion. High levels of bilirubin are associated with decreased risk of coronary heart disease (CHD) and cardiovascular disease (CVD) [1]. While the full spectrum by which bilirubin acts to protect against CVD is not fully understood, there has been evidence of protecting against oxidative stress by reducing reactive oxygen species and possibly having additional anti-atherogenetic properties [2,3]. Previous studies have reported associations of serum bilirubin levels to cardiovascular disease risk factors, including total cholesterol and blood pressure [4-6]. Serum bilirubin levels have also been associated with socioeconomic and behavioral CVD risk factors such as smoking and alcohol intake [7,8]. However, the nature of these associations is unclear, including the potential for residual confounding among serum bilirubin levels and the associated CVD risk factors due to factors measured poorly or not measured at all. Notably, the temporal ordering of variation in these variables is hard to determine, raising challenges in separating 'cause' from 'effect'.

Genotype is uniquely unaffected by most other epidemiologic risk factors. If one accepts the notion that genotypes are in a sense "randomly assigned" during gamete formation, then using genetic variation as an independent variable is attractive. Relationships between genotype and outcomes have only limited susceptibility to confounding and in particular are generally not subject to temporal misspecification of 'cause' and 'effect' (i.e., reverse causality). Thus, genetic variation can be used to aid in inferring causality between a gene product and some outcome of interest. If a genetic variant is strongly causal of a measured exposure, the variant can be used as a proxy for exposure during modeling. This approach, known as Mendelian randomization, can provide evidence of causal inference between two variables [9]. Mendelian randomization has particular utility for evaluation of biomarkers that are highly influenced by a single gene, are of interest in a disease process, and are highly subject to confounding.

The gene UGT1A1 codes for a liver-specific glucoronosyl transferase that converts bilirubin into a more water-soluble form the body is better able to excrete. Homozygosity at the polymorphic promoter repeat locus UGT1A1*28 leads to decreased ability of the UGT1A enzyme to metabolize bilirubin and subsequent mild hyperbilirubinuria (also known as Gilbert's syndrome) [10-12]. Due to UGT1A1*28's strong causal link to bilirubin, use of the UGT1A1*28 genotype to more precisely assess bilirubin - CVD risk factor associations is an attractive and informative application of the Mendelian randomization approach.

In this study, we describe use of the Mendelian randomization approach to evaluate relations of serum bilirubin levels with CVD risk factors and subclinical atherosclerosis in a sample of relatively healthy Old Order Amish individuals from Lancaster County, PA. We subsequently contrast the observed crude estimates with those obtained utilizing Mendelian randomization to illustrate its use. Separating causal from non-causal relations provides evidence of bilirubin's mechanistic role in reducing CVD.

## Methods

### Study sample

The HAPI Heart Study began recruitment in 2003 with the goal of identifying genes that interact with environmental exposure to alter risk of CVD. The study was carried out in an Old Order Amish community in Lancaster County, PA, and the study sample included 868 individuals aged 20 years and older who were relatively healthy. Details of the study aims and recruitment procedures have been previously described [13]. Exclusion criteria included severe hypertension (blood pressure > 180/105 mmHg), malignancy, and kidney, liver or untreated thyroid disease. The study protocol was approved by the Institutional Review Board at the University of Maryland, School of Medicine and informed consent was obtained from each study participant.

Physical examinations were conducted at the Amish Research Clinic in Strasburg, PA. Anthropometric variables, including height, weight, and blood pressure, were assessed by study personnel. A fasting blood sample was obtained for measurement of cholesterol and triglyceride levels. Biochemical assays were performed by Quest Diagnostics (Baltimore, MD). We also measured resting brachial artery diameter. Brachial artery diameter was measured using a linear array ultrasound utilizing a previously described technique [14,15]. Briefly, the left brachial artery was imaged above the antecubital fossa in the longitudinal plane by continuous 2D gray-scale imaging with an 11 mHz ultrasound (Phillips HDI 5000CV). Using guidelines established by the International Brachial Artery Reactivity Task Force [16], all subjects were fasting overnight and none were on any vasoactive medications. The diameter of each artery was measured in a blinded fashion by a trained technician. High resolution B-mode ultrasound was carried out to image the right and left common carotid arteries. IMT was measured between lumen intima and media-adventitia interfaces of the far wall by a single reader using an automated edge detection system.

As part of the study protocol, participants underwent a cold pressor test (CPT). While resting in supine state, participants submerged their right hand and wrist to the ulnar styloid in ice water for 2.5 min. Blood pressure response to the stimulus was measured by calculating the area under the curving using measurements of both systolic and diastolic blood pressure at baseline (pre-emersion), 1 min and 2.5 min.

### Genotyping of UGT1A1*28 locus

The UGT1A1*28 locus was genotyped using the method of Borlak et. al. [17] with slight modification to accommodate the Roche LightCycler 480. PCR was performed on 3 ng of genomic DNA with 2 ul of Lightcycler 480 Genotyping Master in a total reaction volume of 10 ul. All other reagents and concentrations were as per Borlak; PCR conditions were: denaturation (95°C for 5 min), thermocycling for 45 cycles (95°C for 10 s, 56°C for 20 s, 72°C for 20 s), single acquisition and (95°C for 2 min, 40°C for 2 min, 75°C continuous, 1 acquisition per °C). Two alleles were present at this locus in the Amish sample, the 6 allele and the 7 allele, with allele frequencies of 0.57 and 0.43 respectively. The genotype frequencies were slightly out of Hardy Weinberg equilibrium (*p *= 0.04) due to an over representation of heterozygotes.

### Analytic approach

#### Mendelian randomization

The Mendelian randomization approach exploits the fact that genotype precedes life events and is therefore not affected by lifestyle, socioeconomic or any factors that follow conception. To the extent that alleles are randomly assigned at gamete formation, genotype may be thought of analogously to assigned treatment in randomized trials. As previously reviewed [9], Mendelian randomization is an application of instrumental variable analysis, and with certain assumptions the genotype-phenotype relation can be utilized to attain un-confounded estimates of the relation between the gene product and outcomes of interest. These assumptions include an adequately strong relation between genotype and phenotype and the absence of alternate pathways from genotype to the outcome of interest (e.g. pleiotropy, population stratification, linkage disequilibrium). The first assumption--a strong relation between genotype and phenotype--has been previously demonstrated for UGT1A1*28 and bilirubin levels [10-12] and is reflected in our data by the significant differences in bilirubin levels by genotype. As long as these remaining assumptions are met (see below), using genotype as the exposure in analysis will be analogous to modeling assigned treatment in an intention to treat analysis.

#### Causal assumptions

**Figure 1:**
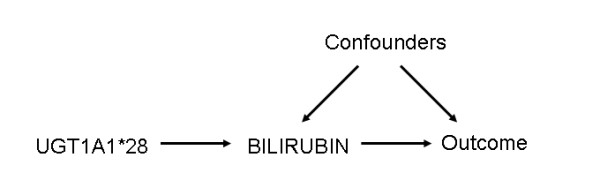
**A diagram representing assumed relations in the causal system**. In this figure, the relation between bilirubin and CVD risk factors is of interest, but is potentially confounded by measured or unmeasured factors. Genotype at the UGT1A1*28 locus affects CVD risk factors only through its relation with bilirubin and is not subject to confounding. The CVD risk factors listed in Table 1 were considered.

Figure 1 is a causal diagram portraying the assumptions that underlie the relations among UGT1A1*28 genotype, serum bilirubin levels and CVD risk factors. The true causal relationship between serum bilirubin levels and CVD risk factors is of interest, but this relationship may be confounded by factors, either measured or unmeasured, as shown in Figure 1. These factors represent causes of both the exposure and outcome, and failure to appropriately address confounding will distort the relation of interest [18,19]. One can see that the relationship between UGT1A1*28 and CVD risk factors are not effected by the confounders. Under the assumption that UGT1A1*28's affect is primarily mediated by bilirubin, it's causal effect can be estimated. Any potential effect of the UGT1A1 enzyme to glucuronidate other substance affecting our outcome measures, even if limited, cannot be accounted for in this model.

### Statistical methods

The principle of Mendelian randomization is illustrated by contrasting the correlations observed between serum bilirubin levels and CVD risk factors without consideration for UGT1A1*28 genotype with the presumed unconfounded results obtained between serum bilirubin levels and CVD risk factors using a two-stage approach that utilizes the genotype ~ outcome and genotype ~ bilirubin regressions [20]. The simple (potentially confounded) estimates were obtained by regressing serum bilirubin levels (the independent variable) on each CVD risk factor (the dependant variable) separately. The Mendelian randomization estimate of the serum bilirubin-CVD risk factor correlation was obtained by dividing the regression coefficient representing the genotype-CVD risk factor effect by the regression coefficient representing the genotype-serum bilirubin effect.

All of the participants of the HAPI Heart Study are related. To address the correlations potentially existing in phenotype by virtue of the fact that subjects are related, we accounted for residual familial correlations in phenotype using a variance component regression framework. Specifically, we modeled variation in the trait as a function of fixed covariates, a polygenic component and a normally distributed error component. The polygenic component was derived from the kinship coefficient matrix which describes the relationship of each pair of individuals in the sample. The software package SOLAR was used for the analysis (Southwest Foundation for Biomedical Research, San Antonio, TX). Estimates of associations of CVD risk factors with bilirubin and with UGT1A1*28 genotype were evaluated using this variance components approach. Bootstrap sampling was used to estimate empirical 95% confidence intervals for the Mendelian randomization estimates. Since the existing literature shows that elevated bilirubin was associated with decrease CVD, we a priori tested for measured CVD risk factors within our cohort, as listed in Table 1. These selected CVD risk factors are highly correlated, so a strict bonferroni correction would potentially result in excessive false negatives. Therefore we elected to report uncorrected *p*-values.

**Table 1:** Observational estimates (95% confidence interval) of the phenotype-bilirubin association and the corresponding variance components *p*-value, mean phenotype value (standard deviation) and significance test of mean difference comparing 7/7 homozygotes with carriers of the 6 allele and point estimates and 95% confidence interval for estimates of effect using Mendelian randomization

	Traditional Approach		Mendelian Randomization Approach				
	
Trait	Estimate (95% CI)	*p*	6/6 mean (SD)	6/7 mean (SD)	7/7 mean (SD)	7/7 vs 6 carriers *p*	Estimate (95% CI)
Body Mass Index (kg/m2)	-1.5 (-2.4, -0.5)	0.003	26.6 (4.7)	26.5 (4.3)	27.0 (4.6)	0.43	0.6 (-0.5, 1.6)
Waist Circumference (cm)	-1.9 (-4.2, 0.4)	0.11	87.5 (11.2)	86.9 (10.8)	88.0 (11.1)	0.16	2.4 (0.0, 5.2)
SBP (mmHg)	-2.8 (-5.9, 0.3)	0.08	122.4 (14.3)	120.9 (15.4)	120.6 (13.5)	0.50	-1.9 (-4.8, 1.9)
DBP (mmHg)	-1.7 (-3.5, 0.1)	0.07	77.7 (8.5)	76.2 (8.6)	76.4 (8.9)	0.74	0.7 (-2.6, 1.8)
Heart Rate (/s)	-0.9 (-3.0, 1.3)	0.43	79.0 (8.5)	78.0 (7.8)	78.4 (8.9)	0.77	-0.5 (-3.1, 2.3)
Triglycerides (mg/dl)	-7.4 (-16.2, 1.4)	0.10	68.4 (42.7)	67.0 (40.5)	71.9 (41.6)	0.39	5.7 (-4.1, 15.8)
Fasting HDL Cholesterol (mg/dl)	-1.4 (-4.4, 1.7)	0.38	56.4 (14.3)	55.6 (14.5)	55.2 (14.9)	0.28	-2.5 (-5.7, 0.7)
Fasting LDL Cholesterol (mg/dl)	-15.9 (-24.8, -7.0)	0.0005	141.0 (44.5)	137.5 (41.7)	139.4 (44.6)	0.62	-3.4 (-13.9, 8.6)
Fasting Total Cholesterol (mg/dl)	-18.6 (-28.3, -8.9)	0.0002	211.1 (48.5)	206.5 (45.9)	209.0 (46.8)	0.54	-4.6 (-15.4, 7.5)
Common Carotid IMT (mm)	-0.03 (-0.06, 0.01)	0.12	0.6 (0.2)	0.6 (0.2)	0.6 (0.2)	0.65	0.0 (-0.03, 0.05)
C Reactive Protein (mg/l)	-0.9 (-1.9, 0.1)	0.07	1.9 (3.8)	2.3 (6.2)	1.7 (2.1)	0.31	-0.8 (-1.4, 0.1)
Pulse Wave Velocity (m/s)	-0.08 (-0.4, 0.2)	0.57	5.3 (1.3)	5.2 (1.1)	5.4 (1.3)	0.17	0.3 (0.0, 0.6)
CPT SBP (mmHg*min)	1.4 (-2.2, 4.9)	0.45	28.8 (17.0)	27.2 (15.6)	31.6 (16.3)	0.02	6.1 (2.1, 10.4)
CPT DBP (mmHg*min)	1.7 (-0.7, 4.2)	0.17	16.5 (11.8)	15.8 (10.5)	18.7 (11.6)	0.01	5.0 (2.4, 8.1)
Flow Mediated Dilation (%)	0.7 (-0.8, 2.1)	0.37	9.7 (5.7)	10.5 (5.8)	11.3 (6.4)	0.12	1.6 (-0.2, 3.2)
Brachial Artery Diameter (mm)	-0.5 (-1.8, 0.7)	0.40	28.2 (5.0)	27.7 (5.4)	26.2 (4.6)	0.003	-1.9 (-2.7, -1.0)

*Abbreviations: CI *confidence interval, *SD *standard deviation, *MR *Mendelian randomization, *CPT *cold pressor test

## Results

### Sample characteristics

The 868 participants of the HAPI Heart Study ranged in age from 20 years to 80 years at the time of the study, and free of any known CVD at that time. Table 2 gives the clinical characteristics of the sample.

**Table 2:** Clinical characteristics (mean (SD)) of the 868 Old Order Amish enrolled in the HAPI Heart Study, Lancaster County PA

Characteristic	Men (n = 460)	Women (n = 408)
Age (yrs)	42.2 (13.6)	45.4 (14.2)
BMI (kg/m^2^)	25.6 (3.2)	27.8 (5.5)
Total Cholesterol (mg/dl)	202.5 (44.3)	215.7 (49.0)
Triglycerides (mg/dl)	63.9 (1.7)	73.8 (45.4)
SBP (mmHg)	121.5 (12.6)	121.4 (16.9)
DBP (mmHg)	77.6 (8.8)	75.8 (8.4)
Diabetes (%)	0.9	1.0
Current Smokers (%)*	20.0	0.0
Lipid Lowering Meds (%)**	1.0	1.0
Antihypertensive Meds (%)**	0.2	0.3

* Indicates use of cigarettes, cigars and pipes

** Medication use assessed at the time of recruitment, before participants were asked to discontinue use

Table 3 shows gender, age and bilirubin levels by UGT1A1*28 genotype. Age was not significantly associated with genotype although a significantly higher percentage of individuals with the 7/7 genotype were male when compared to carriers of the 6 allele (*P *= 0.03). There is no evidence for a role of UGT1A1 in gender determination, and the UGT1A1*28 locus is not linked to the sex chromosomes. Thus, the difference in gender distribution by genotype is most likely not a causal association but rather a chance occurrence; however, since gender differences are also a likely cause of variation of many of the traits further studied, statistical control of gender is warranted. As gender is unlikely to have any causes among other factors considered, statistical adjustment will not result in analysis induced bias, also known as collider stratification bias [21].

**Table 3:** Gender, age and bilirubin levels stratified by UGT1A1*28 genotype of Old Order Amish enrolled in the HAPI Heart Study, Lancaster County, PA

Trait	6/6 (n = 296)	6/7 (n = 392)	7/7 (n = 173)	**7/7 vs**. 6/6-6/7
Gender,% male	58.1%	53.1%	46.2%	0.03
Age, yrs (SD)	44.0 (13.6)	43.0 (14.3)	44.6 (13.7)	0.49
Bilirubin, mg/dL (SD)	0.42 (0.14)	0.49 (0.18)	0.99 (0.42)	< 0.0001

### Association of UGT1A1*28 genotype with serum Bilirubin

As expected, genotype at the UGT1A1*28 locus was strongly associated with bilirubin levels, Table 3. Among those with 7/7 genotype, serum bilirubin level were nearly twice those with 6/7 or 6/6 (*P *< 0.0001 when controlling for gender and age effects and accounting for the expected correlation in the trait due to familial relatedness of the study participants). UGT1A1*28 genotype accounts for 45% of the variation in bilirubin in this population (F-statistic = 682.12). This strong association between genotype and phenotype supports the first condition for use of Mendelian randomization.

### Association of serum Bilirubin levels with CVD risk factors

Simple (i.e., non-Mendelian randomization) associations of serum bilirubin levels with selected CVD risk factors, subclinical atherosclerosis, endothelial function and vascular reactivity were evaluated. Regression coefficients obtained from these analyses, their 95% confidence intervals, and *p*-values evaluating whether these estimates differ from zero are shown in Table 1. Consistent with previous studies showing bilirubin to be inversely associated with CVD and CHD, we observed serum bilirubin levels in this study also to be inversely associated with a variety of CVD risk factors. Specifically, serum bilirubin levels were negatively associated with BMI (*P *= 0.003), LDL cholesterol (*P *= 0.0005) and total cholesterol (*P *= 0.0002). Higher bilirubin levels correlate weakly with lower IMT (*P *= 0.12). These associations (or lack thereof) may be causal or non-causal and may be subject to confounding or reverse causality (i.e., measured bilirubin levels *affected by *the CVD risk factor).

### Association of UGT1A1*28 genotype with CVD risk factors

We hypothesized that genotype would be related to CVD risk factors primarily through its affect on serum bilirubin levels. Mean values (and standard deviation) of the considered CVD risk factors by genotype are presented also in Table 1. Genotype was not associated with either BMI (*P *= 0.43) or waist circumference (*P *= 0.16). Similarly there is no significant difference in blood pressure (SBP *P *= 0.50, DBP *P *= 0.74) or cholesterol levels (HDL *P *= 0.28, LDL *P *= 0.62) across genotype groups. The 7/7 genotype, which corresponds to higher serum bilirubin levels, was associated with a significantly smaller brachial artery diameter (*P *= 0.003) and with cold pressor reactivity (*p *= 0.01).

### Association of serum Bilirubin levels with CVD risk factors: Mendelian randomization approach

The right-most column of Table 1 shows the two-stage estimate (and 95% confidence interval) of the serum bilirubin-CVD risk factor association derived from the Mendelian randomization approach that accounts for UGT1A1*28 genotype. To the extent that Mendelian randomization assumptions are upheld, these estimates may be considered as unconfounded by all measured and unmeasured factors that fit the role of confounders in Figure 1 considered.

Discordance between the simple and Mendelian randomization estimates of the serum bilirubin-CVD risk factor relationship suggests the presence of confounding [22,23]. Some results indicate that confounding is severe enough to reverse the direction of the point estimates, potentially leading to qualitative differences in inference. For example, point estimates of the crude associations of BMI (-1.5, *P *= 0.003), waist circumference (-1.9, *P *= 0.11) and diastolic blood pressure (-1.7, *P *= 0.07) changed direction in the Mendelian randomization analysis (BMI: 0.6, *P *= 0.28; waist circumference: 2.4, *P *= 0.05; DBP: 0.7, *P *= 0.68).

## Discussion

Associations between serum bilirubin and multiple CVD risk factors have been previously reported, but attribution of causality has been challenged by the potential for confounding and uncertainty regarding temporal ordering [24]. Importantly, Lin et. al. [1] utilized genotype at the UGT1A1*28 locus to suggest a causal role for bilirubin in cardiovascular and coronary heart diseases. Most notably, using longitudinal data over 24 years of follow up, they demonstrated a striking unconfounded association with CVD events. While that study supports the hypothesis that increased bilirubin levels cause a decreased risk of CVD, it did not provide estimates of the causal relation of bilirubin with markers of cardiovascular disease burden and hence did not help identify the underlying pathway by which bilirubin might protect against CVD events. In this study, using Mendelian randomization, we attempt to extend previous studies by exploring the association between bilirubin and sub-clinical markers of vascular reactivity and CVD burden in "healthy" subjects, to help delineate potential early mechanistic pathways related to bilirubin. Of note, we find an association between bilirubin and brachial artery diameter and cold pressure reactivity, while utilizing genotype as an instrumental variable per Mendelian Randomization. This association between bilirubin and brachial artery diameter was not present in our data using the traditional regression approach, most likely due to the presence of confounding factors. In this case, it appears that confounding acts to hide or underestimate a potentially causal association. If one believes UGT1A1*28 locus is a reliable instrument of bilirubin levels, these data provide evidence that bilirubin may act on CVD via mechanism associated with brachial artery diameter.

While baseline brachial artery diameter had traditionally been measured to derive brachial flow mediated dilation measures as a surrogate of endothelial function, several studies have demonstrated an independent association between brachial artery diameter with coronary artery calcification [25], serum uric acid [26], blood pressure, serum lipids, smoking and glucose [27,28], carotid IMT [29], pregnancy [30], severity of chronic heart failure [31], angiographic measures of coronary artery disease [32] and CVD events in a cohort of over 2500 subjects [33]. Brachial artery size is also a determinant not only of both FMD and time to peak FMD, but also of non-endothelial related vasodilatory capacity [34]. These studies clearly demonstrate repeatable and significant associations between brachial artery diameter and both CVD risk factors, outcomes and measures of vascular disease, and suggest that in certain populations, it may be more predictive of CVD than FMD measures. As such, our finding of smaller brachial artery diameter in hyperbilirubinemic subjects is consistent with the significant decrease in CVD disease noted in subjects with Gilbert's syndrome [1,35] and raises a potential new mechanism associated with the CVD protective effects of bilirubin, separate from its described antioxidant and anti-inflammatory properties [24,36]. The CPT, by measuring blood pressure change in response to cold stimulus, has been used as a measure of global sympathetic activity and/or response. Our study shows a modest association between bilirubin and increased CPT reactivity. While several studies have shown that increased reactivity in healthy populations may be associated with increased probability for hypertension, the results have not always been consistent, notably in healthy cohorts such as in this study [37,38]. One reason for this could be that increased CPT reactivity may reflect either increased sympathetic activity, which is likely not protective of CVD or may be due to increased reactivity of blood vessels to sympathetic stimulus, which may be a reflection of healthy vasculature and less CVD. Indeed, ageing and obesity are associated with decreased vascular response to sympathetic activity and norepinephrine [39-41]. As such, it is plausible that higher bilirubin levels may be associated with increased vascular response capacity as opposed to an increase in sympathetic neural activity. The lack of association between the UGT1A1*28 genotype and heart rate also suggests no direct increase in baseline sympathetic activity. Mechanistic pathways for the well noted observation of smaller brachial artery diameter with decreased measures of CVD have not yet been elucidated. We did not find an association between the UGT1A1*28 genotype and markers of more advance vascular pathology such as PWV or IMT. This could be secondary to the absence of any such association or also because we studied a relatively young and healthy population with a low CVD disease burden.

## Conclusions

In conclusion, we have utilized Mendelian randomization techniques to address confounding in the estimation of bilirubin's causal relationship with known CVD risk factors. Our data suggest that many of the associations from observational epidemiology studies reported in the literature are subject to un-accounted for confounding and/or reverse causality. Of the tested risk factors, brachial artery diameter and to a lesser degree CPT reactivity were significantly associated with genotype at the UGT1A1*28 locus, providing compelling evidence that bilirubin affects CVD through pathways associated with artery size such as vasomotor tone, reactivity and possibly arterial wall structure.

## Abbreviations

CVD: Cardiovascular Disease; CHD: Coronary Heart Disease; IMT: Intima-media Thickness; FMD: Flow Mediated Dilation; CPT: Cold Pressor Test.

## Competing interests

The authors declare that they have no competing interests.

## Authors' contributions

PFM participated in the design of the study, performed the statistical analysis and drafted the manuscript. BWW participated in the design of the study. KT performed the genotyping. BDM, ARS conceived of the study and oversaw data collection. AP interpreted the results and participated in drafting the manuscript. All authors read and approved the final manuscript.

## Pre-publication history

The pre-publication history for this paper can be accessed here:

http://www.biomedcentral.com/1471-2261/12/16/prepub
